# CRISPR-Cas9 genetic screen leads to the discovery of L-Moses, a KAT2B inhibitor that attenuates Tunicamycin-mediated neuronal cell death

**DOI:** 10.1038/s41598-023-31141-6

**Published:** 2023-03-09

**Authors:** Sofia Pavlou, Stefanie Foskolou, Nikolaos Patikas, Sarah F. Field, Evangelia K. Papachristou, Clive D’ Santos, Abigail R. Edwards, Kamal Kishore, Rizwan Ansari, Sandeep S. Rajan, Hugo J. R. Fernandes, Emmanouil Metzakopian

**Affiliations:** 1 UK Dementia Research Institute, Department of Clinical Neurosciences, Cambridge Biomedical Campus, University of Cambridge, Cambridge, CB2 0AH UK; 2 Open Targets, Wellcome Genome Campus, Hinxton, Cambridge, CB10 1SA UK; 3 Cancer Research UK Cambridge Institute, University of Cambridge, Li Ka Shing Centre, Robinson Way, Cambridge, CB2 0RE UK

**Keywords:** Cell death in the nervous system, Endoplasmic reticulum, Transcriptomics, Proteomics, Functional genomics

## Abstract

Accumulation of aggregated and misfolded proteins, leading to endoplasmic reticulum stress and activation of the unfolded protein response, is a hallmark of several neurodegenerative disorders, including Alzheimer’s and Parkinson’s disease. Genetic screens are powerful tools that are proving invaluable in identifying novel modulators of disease associated processes. Here, we performed a loss-of-function genetic screen using a human druggable genome library, followed by an arrayed-screen validation, in human iPSC-derived cortical neurons. We identified and genetically validated 13 genes, whose knockout was neuroprotective against Tunicamycin, a glycoprotein synthesis inhibitor widely used to induce endoplasmic reticulum stress. We also demonstrated that pharmacological inhibition of KAT2B, a lysine acetyltransferase identified by our genetic screens, by L-Moses, attenuates Tunicamycin-mediated neuronal cell death and activation of CHOP, a key pro-apoptotic member of the unfolded protein response in both cortical and dopaminergic neurons. Follow-up transcriptional analysis suggested that L-Moses provided neuroprotection by partly reversing the transcriptional changes caused by Tunicamycin. Finally, L-Moses treatment attenuated total protein levels affected by Tunicamycin, without affecting their acetylation profile. In summary, using an unbiased approach, we identified KAT2B and its inhibitor, L-Moses, as potential therapeutic targets for neurodegenerative diseases.

## Introduction

A shared pathology between various neurodegenerative diseases is the accumulation of misfolded and aggregated proteins in the brain, leading to cellular dysfunction, loss of synaptic connectivity and eventually neuronal death^1^. However, distinct proteins, brain regions and neuronal subtypes are affected across different neurodegenerative diseases. For example, Alzheimer’s disease (AD) is associated with accumulation of misfolded and aggregated amyloid beta (Aβ) and tau^1^, with the neocortex and hippocampus being the most affected areas^2^. Parkinson’s disease (PD), on the other hand, is characterized by the presence of misfolded and aggregated alpha-synuclein^1,3^ and the dopaminergic neurons within the substantia nigra are the most vulnerable cell type^2^.

Accumulation of misfolded proteins leads to endoplasmic reticulum (ER) stress^4^. To alleviate ER stress and restore homeostasis, cells undergo a rapid and highly coordinated signalling cascade, known as the unfolded protein response (UPR). The UPR activates apoptosis if the damage is irreversible. C/EBP-Homologous protein (*CHOP*, also known as DNA Damage Inducible Transcript 3—*DDIT3*) is a key pro-apoptotic gene, activated by UPR^5^. Emerging evidence indicate a key role for ER stress and UPR in the pathophysiology of protein misfolding disorders^4,6,7^. Therefore, identifying novel modulators of ER stress that dampen the UPR has great therapeutic potential.

CRISPR-Cas9 mediated genetic screens allow the systematic identification of modulators of a specific process. Here, we performed a loss-of-function genetic screen to identify genes involved in Tunicamycin (Tun)-mediated neuronal death of human iPSC-derived cortical neurons. Tun blocks the initial step of glycoprotein biosynthesis within the ER, resulting in accumulation of unfolded glycoproteins and subsequently ER stress^8^ and is therefore widely used to induce ER stress in tissue culture systems.

Here, we performed a genetic screen using a sgRNA library against the druggable genome^9^ to identify genes, whose loss-of-function attenuates Tun-mediated neuronal death. We validated our hits in an arrayed format and identified 13 genes, whose knock-out provided neuroprotection against Tun. L-Moses, a Lysine Acetyltransferase 2B (KAT2B) inhibitor^10^, reduced neuronal cell death, ER stress and UPR activation in both cortical and dopaminergic neurons. To our knowledge, this is the first time L-Moses was shown to be neuroprotective and to have therapeutic potential for both AD and PD.

## Materials and methods

### Cell culture

NGN2-OPTi-OX was a kind donation by the Kotter lab at the University of Cambridge, where the original iPSC line was sourced from the University of Cambridge (https://hpscreg.eu/cell-line/CAMi014-A)^11^. A second NGN2-iPSC line^12^ was sourced by the Ward lab at NIH, as a kind donation. KOLF-2 were sourced by the HipSci consortium and the Wellcome Sanger Institute (https://www.hipsci.org/lines/#/lines/HPSI0114i-kolf_2).

Human iPSCs NGN2-OPTi-OX cells were maintained and cortical-like iNeurons were induced as described before^13^. Tun-treatment was performed on day 14 (d14). A second independent cell line, engineered in the Ward lab^12^, was used to validate the effect of L-Moses.

Human iPSCs (KOLF-2 or NGN2-OPTi-OX) were differentiated into dopaminergic neurons as previously described^14^. Tun-treatment was performed on d32–d35 neurons.

HEK293FT cells were cultured in DMEM/F12 supplemented with 10% FBS and 1% Pen/Strep.

### iPSC engineering

*Cas9* was inserted upstream of *GAPDH*, by CRISPR-mediated homology-directed repair as previously described^15^. Several clones were tested, and one was selected for all experiments, based on its Cas9-editing efficiency. Editing efficiency was assessed by transducing d4 neurons with a lentiviral vector encoding BFP, GFP and a sgRNA for GFP. Neurons were dissociated on d8 and subjected to flow cytometry using CytoFLEX S (Beckman Coulter). Editing efficiency was calculated by comparing the percentage of BFP^+^/GFP^-^ (edited) to BFP^+^/GFP^+^ (total transduced) cells using FlowJo version 10.8.1 Software for macOS (BD Life Sciences).

Tag-RFP was inserted downstream of *CHOP*, separated by a T2A as described previously^15^. The following sgRNA sequence was used: 5ʹ-UGCUCCCAAUUGUUCAUGCU-3ʹ (Merck). Several clones were genotyped by PCR (primers: TATCTTCATACATCACCACACCTGA and TTCTAAAACACATCAGAGATTGGGG) and Sanger sequencing. Validation experiments were performed with two homozygote (518/535) and two heterozygote (403/407) clonal lines. All other experiments were performed with both homozygote lines.

### Drug treatments

Tun was acquired as a ready-made solution in DMSO (SML1287-1ML, Merck). IOWH032 (#S7329, Selleckchem) was dissolved in DMSO; GR144053 (#1263, Tocris) and L-Moses (#6251, Tocris) were dissolved in dH_2_O as per manufacturer instructions. D14 neurons were treated with 100 nM Tun, unless otherwise stated. Inhibitors were added two days before Tun-treatment onset. For the BRB-seq and Mass spectrometry experiments, 12.5 or 25 μM L-Moses was used for cortical or dopaminergic neurons, respectively.

Viability was measured by MTS assay (#ab197010, Abcam) according to manufacturer’s instructions.

### CRISPR-Cas9 screen

A druggable genome sgRNA library (Supplementary Table S1) was lentivirally transduced into d4 neurons. Following 7-day Tun treatment, surviving neurons were collected and processed for sequencing. MAGeCK RRA^16^ was used to perform gene essentiality and enrichment inference. See Supplementary Methods S1 for detailed protocol.

### Quantitative real-time PCR (qPCR)

RNA was extracted using RNeasy kit (Qiagen), cDNA synthesis was performed using qScript cDNA synthesis kit (Quantabio) and qPCR using Luna Universal qPCR master mix (NewEngand BioLabs) and QuantStudio 5 Real-Time PCR system (ThermoFisher Scientific). Taqman assay ID and primer sequences are shown in Supplementary Table S1. Samples were analysed in technical triplicates and normalized to 18S. Results were analysed with the ΔΔCt method and shown as fold change.

### Bulk RNA barcoding sequencing (BRB-seq)

BRB-seq was performed using the MERCURIUS™ BRB-seq kit (Alithea Genomics)^17^. See Supplementary Methods S1 for detailed protocol.

### Western blot

Total protein was extracted in RIPA buffer supplemented with 10% proteinase inhibitor cocktail (Merck) and quantified using Pierce BCA protein assay kit (ThermoFisher Scientific). For KAT2B western, NE-PER Nuclear and Cytoplasmic extraction reagents (ThermoFisher Scientific) were used to enrich for nuclear proteins. PVDF membranes were incubated overnight with 1/1000 Cas9 (#14697, Cell Signalling)^18^, 1/2000 B-Actin (#ab8227, Abcam)^19^, 1/10,000 GAPDH (Abcam, ab128915)^20^, 1/100 KAT2B (#sc-13124, Santa Cruz Biotechnology)^21^, followed by 1 h incubation with HRP-conjugated secondary antibodies. Membranes were developed using SuperSignal West Pico PLUS Chemiluminescent substrate (ThermoFisher Scientific) and bands detected using iBright CL1000 (Invitrogen).

### Liquid chromatography and mass spectrometry

Acetylome and whole proteome analyses were performed on Dionex UltiMate 3000 UHPLC system coupled with nano-ESI Fusion-Lumos (Thermo Scientific) mass spectrometer. See Supplementary Methods S1 for detailed protocol.

### Statistical analysis

Data are presented as mean ± standard error (SEM). One-way ANOVA followed by Dunnett’s or Tukey’s multiple comparisons tests were performed using GraphPad Prism version 9.3.1 for macOS (GraphPad Software). P-value (p) < 0.05 was considered statistically significant.

## Results

### Genetic screen identifies modulators of Tun-mediated neuronal death

To identify genes that ameliorate neuronal cell death caused by Tun-induced ER stress, we performed a loss-of-function pooled genetic screen using our druggable genome sgRNAs library.

**Figure 1:**
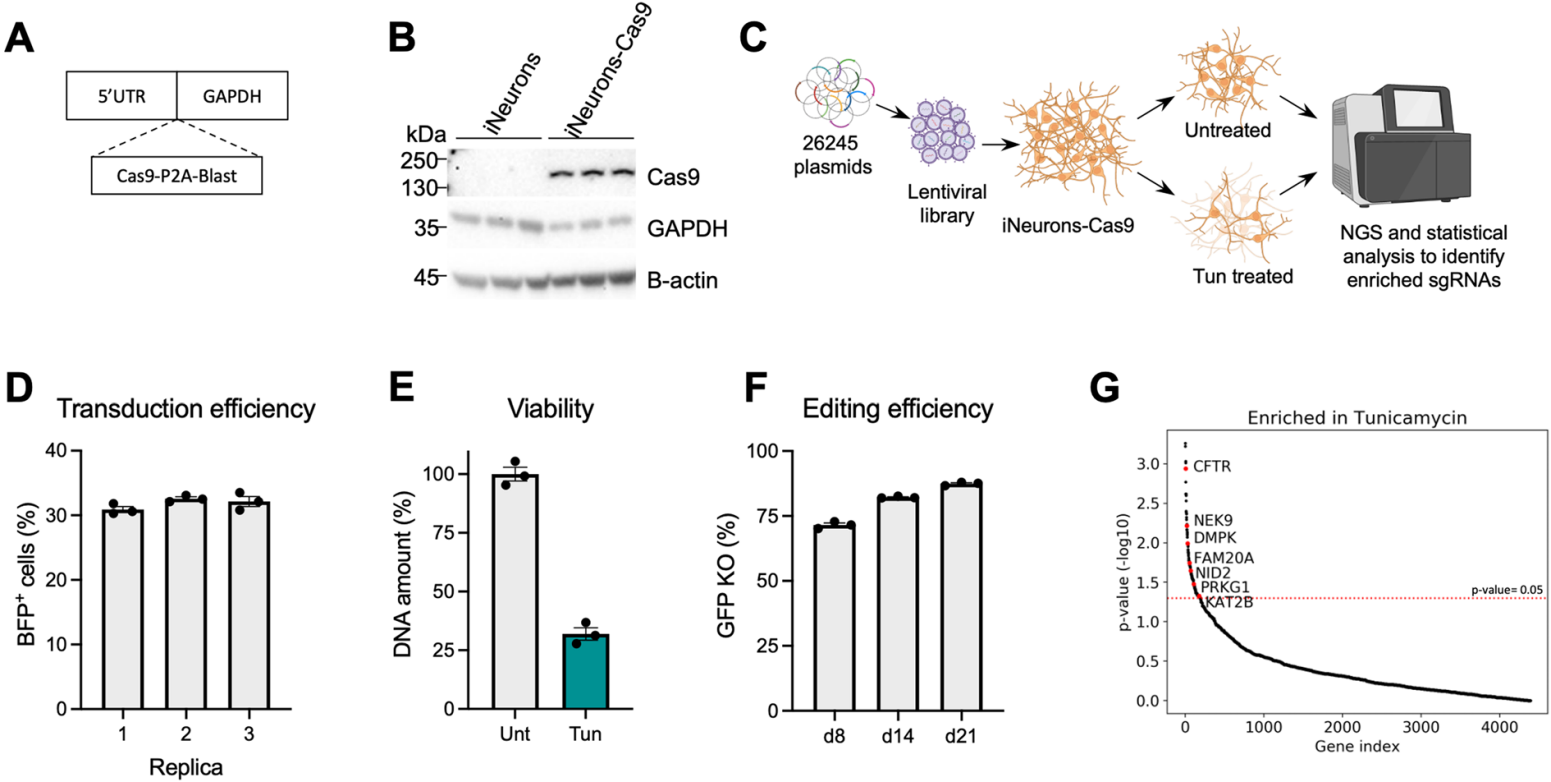
CRISPR-Cas9 genetic screen identifies genes that attenuate Tun-mediated neurodegeneration. (**A**) Cas9 gene was inserted upstream of the endogenous GAPDH. (**B**) Cas9 expression was detected on d4 neurons. GAPDH and B-Actin are shown as controls. Original blots are presented in Supplementary Fig. S7. (**C**) Diagram showing the strategy for the screen. (**D**) Transduction efficiency of the three replicates. (**E**) Viability of Tun-treated samples (100 nM Tun for 7 days) was about 70%, as judged by quantifying the total DNA of the samples. (**F**) Editing efficiency of iNeurons-Cas9. (**G**) Gene hits ranked based on their p-value. Red line indicates p-value of 0.05. Data are shown as mean ± SEM, n = 3.

We first generated a clonal iPSC line that expresses Cas9 under the *GAPDH* promoter (Fig. 1A). We showed that Cas9 was expressed in d4 cortical-like iNeurons by western blot (Fig. 1B). The presence of Cas9 did not affect iNeuron differentiation as determined by qPCR of key pluripotency and neuronal markers (Supplementary Fig. S1A).

Next, we generated a druggable genome sgRNA library that consists of 26,306 sgRNAs targeting 4401 genes and 1000 control non-targeting sgRNAs. The 4401 genes were identified by Finan et al.^9^ and include targets of approved and clinical-phase drugs, genes with similarity with approved drug targets, secreted and extracellular proteins and members of key druggable gene families like G-protein-coupled receptors, kinases and ion channels. The library was sequenced, and the distribution of sgRNAs is shown in Supplementary Fig. S1B.

For the pooled screen (Fig. 1C), d4 neurons were transduced with the sgRNA library at a MOI of 0.3 (Fig. 1D) and d14 neurons were treated with 100 nM Tun, a glycoprotein synthesis inhibitor that results in ER stress. At this concentration and after 7-day treatment, Tun resulted in approximately 70% cell death (Fig. 1E). The editing efficiency was determined by transducing a lentiviral vector encoding BFP, GFP and a sgRNA against GFP into d4 neurons. As shown in Fig. 1F, editing was about 71% in d8 neurons, and increased to 82% in d14 (onset of Tun treatment) and 87% in d21 (day of sample collection). We collected the surviving neurons on d21 and following PCR amplification and sequencing of the sgRNAs, we identified 188 genes (Supplementary Table S2), whose sgRNAs were significantly enriched in the Tun sample when compared to the untreated (Unt) sample (Fig. 1G). Enrichment analysis for Gene Ontology Molecular Function shows that the 188 hits are involved in protein kinase activity, receptor ligand activity and ion binding among others (Supplementary Fig. S1B). In terms of Biological Processes, the identified hits are enriched for protein phosphorylation, neuropeptide signalling pathway, intrinsic apoptotic signalling pathway in response to DNA damage and positive regulation of neuron projection development (Supplementary Fig. S1C).

### Arrayed screen validated identified hits

**Figure 2:**
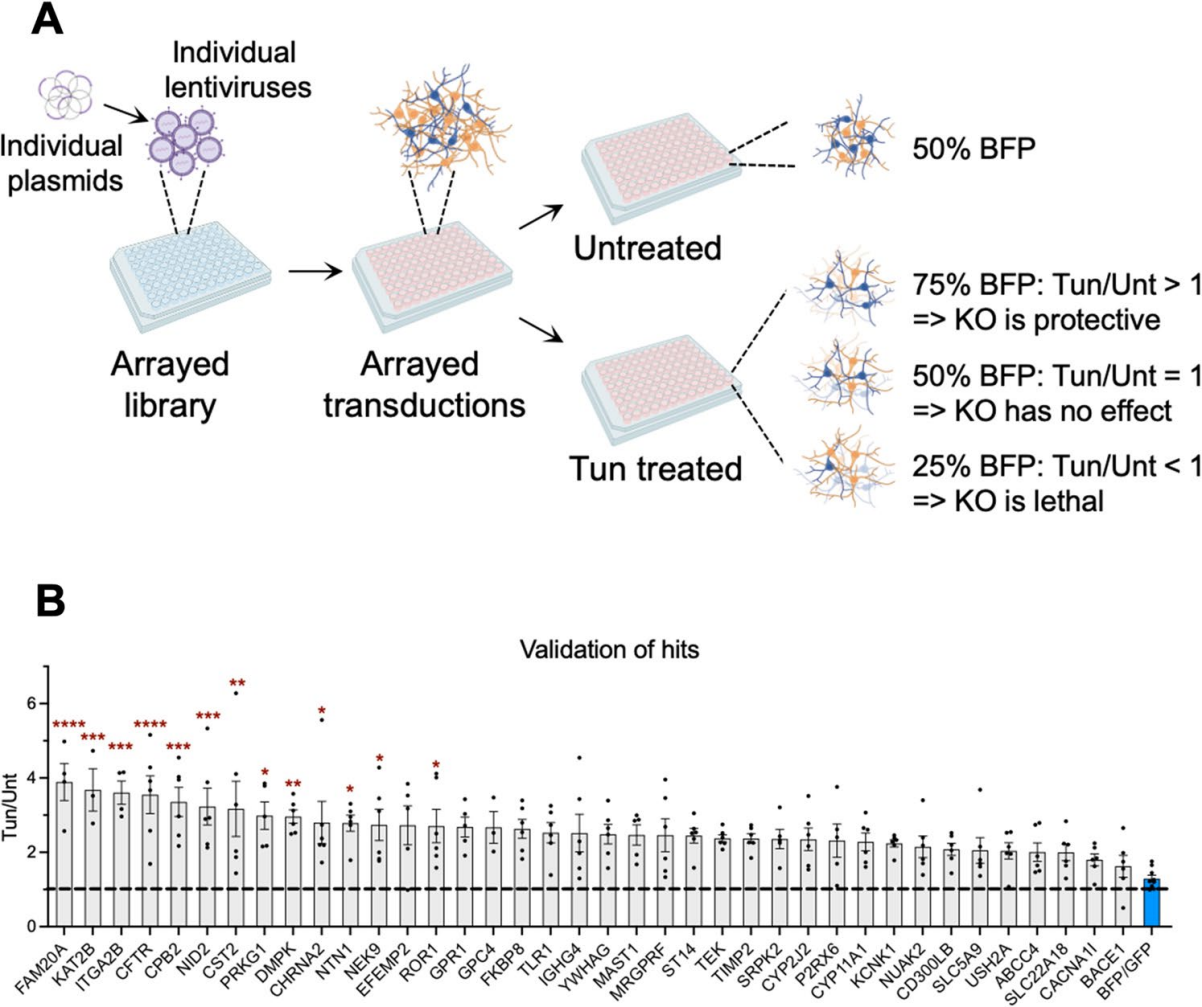
Arrayed screen identifies 13 significant hits. (**A**) Diagram showing the strategy for the arrayed screen. In this simplified example, transduction efficiency is 50%. In the actual experiment, transduction efficiency varied between the different KOs. (**B**) Graph showing the Tun/Unt ratio of BFP positive cells. D14 neurons were treated with 100 nM Tun for 7 days. Data are shown as ± SEM, n = 6 (3 replicas from 2 independent experiments). Dotted line shows Tun/Unt = 1. One-way ANOVA with Dunnett’s multiple comparisons test. *p < 0.05, **p < 0.01, ***p < 0.001 and ****p < 0.0001 compared to the neutral control (BFP/GFP; blue bar).

To validate our pooled screen in an arrayed format (Fig. 2A), we selected 38 genes that had at least three significantly enriched sgRNAs. We transduced d4 iNeurons-Cas9 with a pool of three sgRNAs for each gene in an arrayed format, so that each well contained knockout (KO) cells of one gene. Transduced cells were then split into two groups: Unt and Tun. Transduction efficiency was different between the different KOs as well as the two experimental replicas (the lentivirus production and transduction was performed twice). Four days post transduction, the average transduction efficiency was 25.44% (min = 8.01% and max = 56.9%) for the first experiment and 28.3% (min = 10.3% and max = 65.7%), as assessed by the percentage of BFP positive cells by flow cytometry. On d14, 10 days post transduction, Tun group was treated with 100 nM Tun for 7 days, to mimic the pooled screen conditions. On d21, the percentage of BFP positive cells, as an indicator of the percentage of KO cells, in the Tun versus Unt samples was determined by flow cytometry. All our hits had a Tun/Unt ratio of > 1, and 13 of them were significantly neuroprotective when compared to the control (Fig. 2B and Supplementary Table S3).

Three of our validated hits (PRKG1, DMPK, NEK9) are serine/threonine protein kinases and FAM20A is a pseudokinase that acts as an allosteric activator of the protein kinase FAM20C^22^. ROR1 is a tyrosine-protein kinase transmembrane receptor that modulates neurite growth^23^, whereas CPB2 controls the activity of biologically active peptides by cleaving C-terminal arginine or lysine residues^24^. ITGA2B is involved in cell adhesion^25^ and CFTR is associated with cystic fibrosis and involved in ion and water transport^26^. Finally, KAT2B is an acetyltransferase that promotes transcriptional activation^27^.

### L-Moses attenuates Tun-mediated effects in cortical neurons

**Figure 3:**
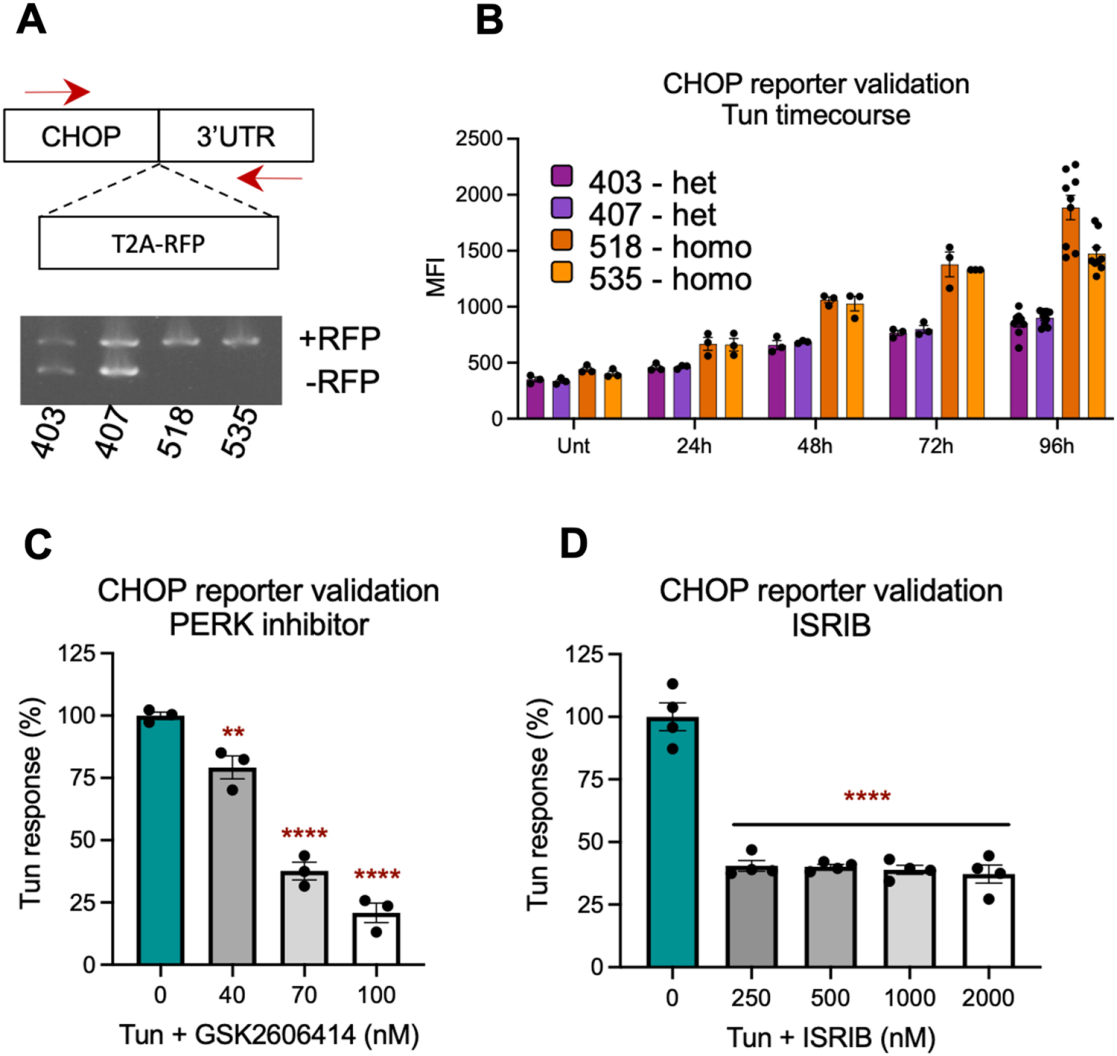
Generation and validation of CHOP reporter clonal cell lines. (**A**) RFP was introduced downstream the CHOP gene, resulting in a shift in the PCR amplicon. Red arrows indicate position of primers. (**B**) Time-course showing increase in RFP expression in iNeurons derived from the four clonal cell lines, following treatment with 100 nM Tun. The two homozygote lines (orange bars) show approximately two-fold higher median fluorescent intensity (MFI) when compared to the heterozygote lines (purple bars). (**C**) The Tun response (100 nM Tun-treated minus Unt MFI) is reduced with increasing concentrations of GSK2606414, a known Tun inhibitor. (**D**) ISRIB, another inhibitor of the pathway, reduced the Tun response. Data are shown as ± SEM, n > 3. One-way ANOVA with Dunnett’s multiple comparisons test. **p < 0.01, ***p < 0.001 and ****p < 0.0001 compared to 100 nM Tun only control (blue bar).

Tun-mediated ER stress leads to activation of the UPR and upregulation of *CHOP*, a pro-apoptotic gene. We hypothesize that inhibition of the identified hits attenuates Tun-mediated neuronal death by directly or indirectly reducing the UPR and therefore CHOP activation. To test our hypothesis, we generated CHOP reporter clonal lines (Fig. 3Α), by inserting Tag-RFP downstream of endogenous *CHOP* in the NGN2 OPTi-OX Cas9 iPSCs. Two heterozygote (403, 407) and two homozygote (518, 535) clones were differentiated into cortical-like iNeurons and subjected to a Tun time-course. As shown in Fig. 3Β, a time-dependent increase in median fluorescent intensity (MFI) indicating an increase in CHOP levels was observed for all four clones. The homozygote clones demonstrated approximately two-fold higher MFI when compared to the heterozygotes, confirming their genotype. To further validate our CHOP reporter lines, we treated iNeurons derived from the two homozygote lines with two known UPR inhibitors: GSK2606414, a PERK inhibitor^28^ and ISRIB, an integrated stress response (ISR) inhibitor that acts downstream of PERK^29^. Both inhibitors reduced Tun-mediated increase in MFI in our CHOP reporters (Fig. 3C,D).

To validate our screen results using an independent approach, we pharmacologically inhibited three of the identified hits: ITGA2B using GR144053, CFTR using IOWH032 and KAT2B using L-Moses. We pre-treated d12 neurons with the different inhibitors, followed by co-treatment with 100 nM Tun from d14 onwards. GR144053 and IOWH032 had no effect on Tun-mediated cell death or CHOP levels, as shown in Supplementary Fig. S2.

**Figure 4:**
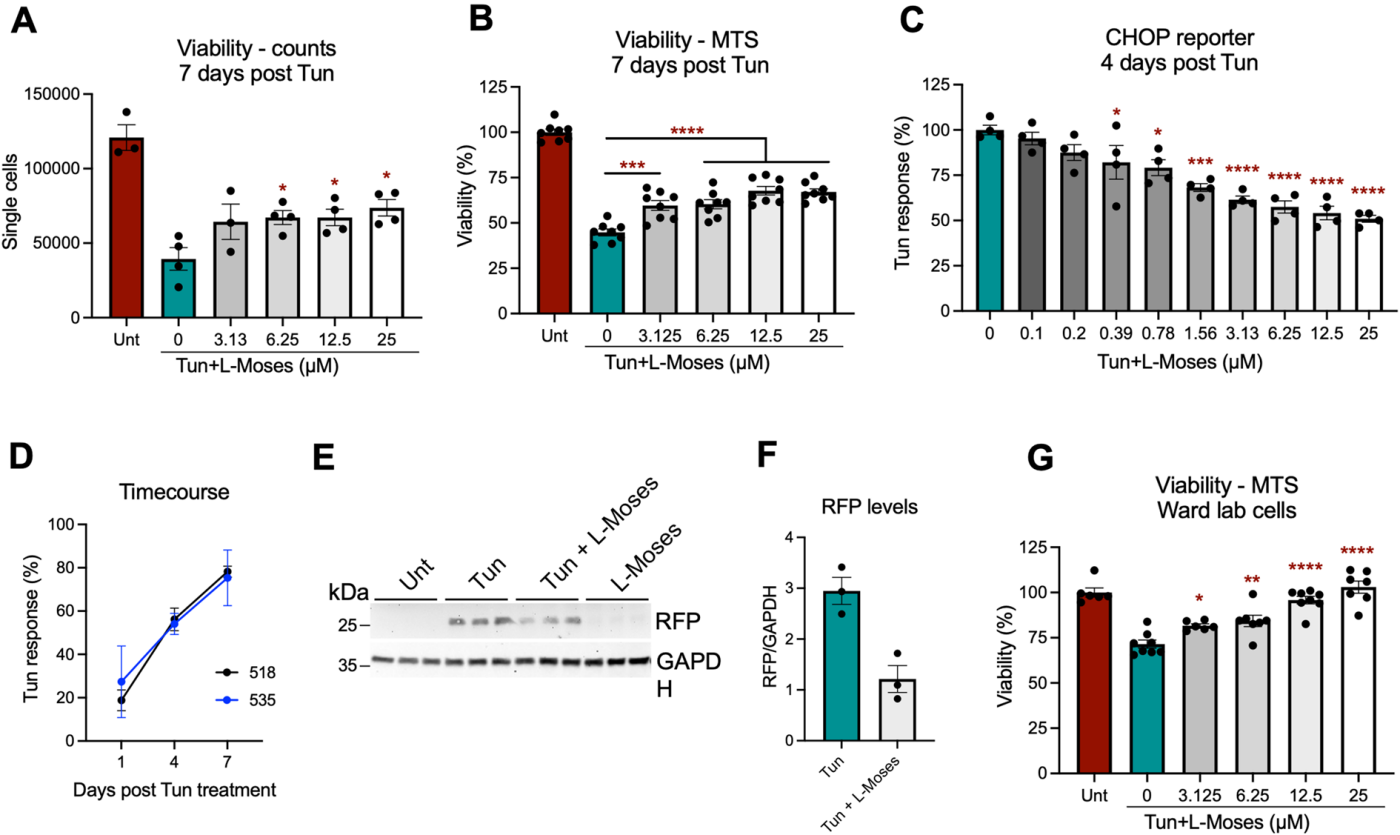
L-Moses attenuates Tun-mediated effects on cortical neurons. (**A**,**B**) Co-treatment with L-Moses significantly increases viability of 100 nM Tun-treated samples, as judged by cell counting (A) and MTS assay (**B**). (**C**) L-Moses reduces Tun-mediated response (Tun-treated minus Unt MFI) in the CHOP reporter lines, in a dose-dependent manner. (**D**) Time-course showing that L-Moses effect is reduced with continuous exposure to 100 nM Tun, using both homozygote clonal lines (518 and 535). (**E**,**F**) Western blot confirming reduces RFP levels in Tun + L-Moses treated samples, when compared to Tun-treated. Original blots are presented in Supplementary Fig. S7. (**G**) Significant rescue in viability, following L-Moses treatment, was observed in cortical neurons derived from an independent iPSC cell line (Ward lab). One-way ANOVA with Dunnett’s multiple comparisons test. *p < 0.05, **p < 0.01, ***p < 0.001 and ****p < 0.0001 compared to 100 nM Tun only control (blue bar).

Treatment with L-Moses, a KAT2B inhibitor, resulted in significant attenuation of Tun-mediated cell death, as determined by cell counts using flow cytometry (Fig. 4A) and MTS viability assay (Fig. 4B). In addition, L-Moses treatment resulted in dose-dependent reduction in CHOP levels after 4 d of Tun treatment (Fig. 4C), as assessed using our CHOP reporter clonal lines. L-Moses reduces Tun-mediated CHOP levels by about 80% after 1 day treatment with Tun, 60% after 4 days and 20% after 7 days (Fig. 4D). Western blot against RFP confirmed the reduction in CHOP-driven RFP levels in neurons co-treated with Tun + L-Moses when compared to Tun (Fig. 4E,F). To confirm the specificity of the results, we repeated the experiment using a second independent doxycycline-inducible neuronal line, developed by the Ward lab^12^, where overexpression of *NGN2* results in glutamatergic cortical neurons. As shown in Fig. 4G, L-Moses also attenuated Tun-mediated cell death in cortical-like neurons derived from this line.

KAT2B expression at both mRNA and protein level was not affected by Tun. A significant increase in KAT2B protein levels was observed in neurons treated with Tun + L-Moses (Supplementary Fig. S3).

In summary, we demonstrated that pharmacological inhibition of KAT2b by L-Moses partly rescued, in a dose-dependent manner, the Tun-mediated neuronal death and CHOP activation observed in cortical-like iNeurons.

### L-Moses partially reverses Tun-mediated transcriptional changes in cortical neurons

**Figure 5:**
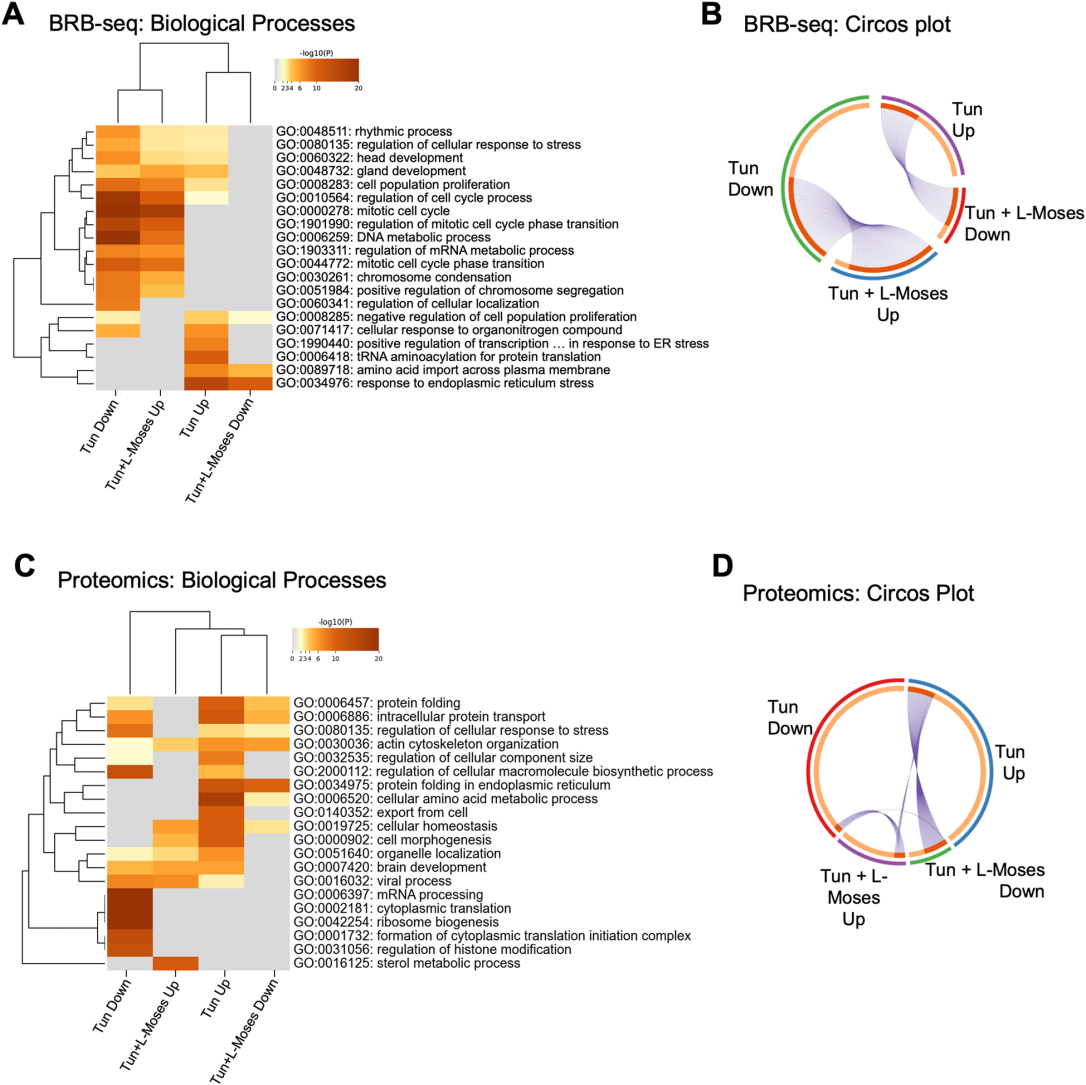
L-Moses attenuates Tun-mediated transcriptional and proteomic changes on cortical neurons. (**A**) Hierarchical clustering of GO Biological Processes terms upregulated or downregulated in Tun versus Tun + L-Moses, as determined by BRB-seq. (**B**) Circos plot highlighting the overlaps (purple lines) between genes upregulated by Tun and downregulated by Tun + L-Moses and vice versa, as determined by BRB-seq. (**C**) Hierarchical clustering of GO Biological Processes terms upregulated or downregulated in Tun versus Tun + L-Moses, as proteomic analysis. (**D**) Circos plot highlighting the overlaps (purple lines) between proteins upregulated by 100 nM Tun and downregulated by Tun + L-Moses and vice versa, as determined by proteomic analysis.

KAT2B is a lysine acetyltransferase acting as a transcriptional activator. We therefore hypothesize that KAT2B, at least partly, regulates the transcriptional changes that follow Tun. Consequently, inhibition of KAT2B by L-Moses would result in the reversal of these KAT2B-mediated transcriptional signatures. To investigate this hypothesis, we performed bulk RNA barcoding sequencing (BRB-seq) (Supplementary Table S4 and Supplementary Fig. S4A)^17^. We found that L-Moses had minimal effect on transcription under basal conditions (13 genes were significantly upregulated and 16 were downregulated). Enrichment analysis for Gene Ontology (GO) Biological Processes terms showed that Tun resulted in upregulation of genes involved in ER stress (Supplementary Fig. S4B). Co-treatment with Tun + L-Moses resulted in downregulation of genes involved in ER stress (Supplementary Fig. S4C). We next looked at the hierarchical clustering of the enriched GO terms that were upregulated or downregulated by Tun or Tun + L-Moses. As shown in Fig. 5A, GO terms downregulated in Tun clustered with GO terms upregulated in Tun + L-Moses and vice versa. We applied the same methodology for GO Molecular Functions and found similar results (Supplementary Fig. S4D). For example, genes related to the unfolded protein binding were significantly upregulated following Tun treatment and downregulated with Tun + L-Moses co-treatment. The fold change differences in expression of genes involved in ER stress, under the four different experimental conditions, are shown in Supplementary Fig. S4E. Circos plot highlights the gene overlaps between the groups: 34 genes upregulated by Tun are downregulated by Tun + L-Moses and 79 genes downregulated by Tun are upregulated by Tun + L-Moses (Fig. 5B).

No significant overlap was observed between the screen hits and the differentially expressed genes, except for MARS (screen hit that was upregulated by Tun), GPC4 (screen hit downregulated by Tun) and GSTP1 (screen hit reduced by Tun and upregulated by Tun + L-Moses).

Together these results suggest that L-Moses, at least partly, reversed the transcriptional changes mediated by Tun in cortical neurons.

### L-Moses attenuates Tun-mediated changes at the protein level, without affecting their acetylation status

We demonstrated that pre-treatment with L-Moses attenuates Tun-mediated transcriptional effects in cortical neurons. Next, we investigated whether this attenuation reflects changes at the protein level by performing proteomics analysis using tandem mass tag (TMT) quantification (Supplementary Table S5). As shown in Fig. 5C,D, and similar to the transcriptomic analysis, proteins that were significantly increased by Tun-treatment, were reduced by co-treatment with L-Moses and vice versa. GO analysis for Biological processes highlighted protein folding and regulation of cellular response to stress among the GO terms that were upregulated in Tun-treated samples and downregulated in Tun + L-Moses.

L-Moses targets the bromodomain of KAT2B^10^, suggesting that it inhibits KAT2B function by interfering with its capacity to regulate gene transcription. Therefore, we hypothesize that the effects of L-Moses are mainly at the transcriptional level. To investigate whether L-Moses affects the acetylome profile of our neurons, we performed full lysine acetylome enrichment analysis using TMT quantification. As shown in Supplementary Fig. S5 and Supplementary Table S6, Tun-treatment significantly attenuated the acetylome of cortical neurons. However, only one significant change (YARS1) is observed in cortical neurons co-treated with Tun and L-Moses when compared to Tun.

These data confirm our hypothesis that L-Moses attenuated neuronal cell death caused by Tun, by interfering with KAT2B’s ability to regulate transcription, but not acetylation of non-histone proteins. This translates to changes in the total levels of proteins, without affecting their acetylation profile.

### L-Moses attenuates Tun-mediated effects in dopaminergic neurons

We demonstrated that L-Moses partially rescues Tun-mediated neuronal cell death in cortical-like neurons, one of the main cell types affected in AD. We next investigated whether L-Moses had similar effects on dopaminergic neurons, the main cell type affected in PD. KOLF2 iPSCs were differentiated into dopaminergic neurons and their identity was confirmed by the expression levels of key dopaminergic markers compared to the cortical-like iNeurons by BRB-seq and immunofluorescence (Supplementary Fig. S6A,B).

**Figure 6:**
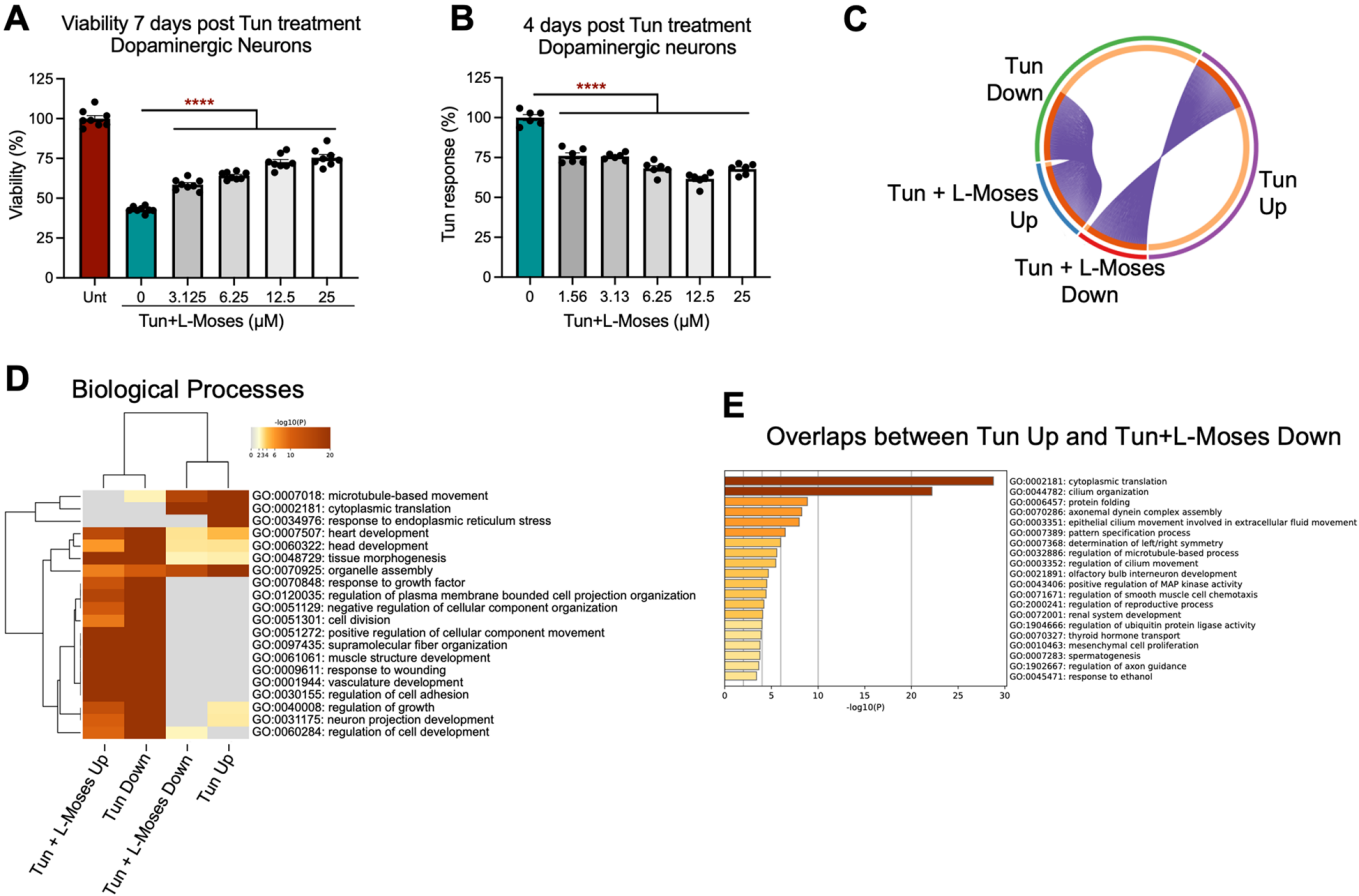
L-Moses attenuates Tun-mediated transcriptional changes on dopaminergic neurons. (**A**) Co-treatment with L-Moses rescues Tun-mediated cell death in a dose dependent manner, as shown by MTS assay. (**B**) L-Moses reduces Tun response (Tun-treated minus Unt MFI) in a dose dependent manner. (**C**) Circos plot showing the overlap (purple lines) between genes upregulated by Tun and downregulated by Tun + L-Moses and vice versa. (**D**) Hierarchical clustering of GO Biological Processes terms upregulated or downregulated in Tun versus Tun + L-Moses. (**E**) GO Biological Processes terms enriched in the genes that overlap between the ones upregulated by 100 nM Tun and downregulated by Tun + L-Moses.

Dopaminergic neurons were pre-treated with L-Moses for 2 d, followed by 7-d treatment with Tun. As shown in Fig. 6A, L-Moses resulted in a dose-dependent increase in viability of dopaminergic neurons, as measured by MTS assay. Using our CHOP-reporter lines, we also demonstrated that L-Moses reduces Tun-mediated CHOP levels in dopaminergic neurons (Fig. 6B).

BRB-seq (Supplementary Table S7) and GO analysis demonstrated that genes involved in ER stress and protein folding were upregulated in Tun and downregulated in Tun + L-Moses conditions (Supplementary Fig. S6C,D). Hierarchical clustering suggested that GO terms upregulated by Tun are downregulated by Tun + L-Moses co-treatments and vice versa (Fig. 6D and Supplementary Fig. S6E). An overlap of 356 genes was observed between genes downregulated by Tun and upregulated by Tun + L-Moses, as shown by circos plot in Fig. 6C. These genes are involved in actin cytoskeleton organization, development, and regulation of growth among others (Supplementary Fig. S6F). The fold change differences in the expression of genes involved in ER stress are highlighted in Supplementary Fig. S6G. Similarly, a significant overlap (326 genes) was observed between the genes upregulated by Tun and downregulated by Tun + L-Moses, shown by circos plot in Fig. 6C. GO analysis of these overlapped genes demonstrated enrichment in cytoplasmic translation, cilium organization and protein folding (Fig. 6E).

Together our data demonstrated that inhibition of KAT2B, identified by a genetic screen in cortical neurons, partly reversed the Tun-mediated neuronal cell death, activation of CHOP and transcriptional changes observed in dopaminergic neurons. Therefore, L-Moses has therapeutic potential in reducing ER stress-mediated defects in both AD and PD.

## Discussion

Accumulation of misfolded proteins, subsequent ER stress and activation of the UPR are hallmarks of the pathology of several neurodegenerative disease, including AD and PD. Here, we performed a druggable genome CRISPR-Cas9 screen to identify modulators of Tun-mediated neuronal death. We identified and validated 13 genes, whose knockout provides neuroprotection. Pharmacological inhibition of KAT2B by L-Moses partially rescued Tun-mediated neurodegeneration, increase in CHOP levels and transcriptional effects in both cortical and dopaminergic neurons. Full proteomic and acetylome analysis of cortical neurons, demonstrate that the total levels of proteins are attenuated by Tun + L-Moses co-treatment compared to Tun alone, but the acetylation profile of the proteins is unaffected.

Genetic screens are proving to be a powerful tool in identifying new modulators of specific processes. Although several genetics screens performed in the past aimed to identify genes involved in different aspects of ER stress and the UPR pathway^30–34^, to our knowledge this is the first screen performed in iPSC-derived neurons aiming to identify knockouts that are neuroprotective against Tun. Due to their non-mitotic nature, the difficulties in delivering large DNA fragments, the increased silencing of Cas9 and their heterogeneity, iPSC-derived neurons are particularly challenging cells for genetic screens. This is highlighted by the limited number of publications regarding screens on neurons when compared to cancerous cell lines. To overcome these challenges, we generated a clonal line, in which Cas9 is driven by the *GAPDH* promoter, a constitutively active gene. In addition, we used an inducible system that results in a relatively homogeneous neuronal population and validated our results in an arrayed format. Finally, we validated our key findings using a second independent iPSC line to eliminate cell line-specific effects.

Our validation arrayed screen led to the identification of 13 genes, whose loss-of-function is neuroprotective against Tun. Further investigation is required to elucidate the possible mechanisms by which inhibition of these genes leads to ER stress resistance. Pharmacological inhibition of ITGA2B using GR144053 and CFTR using IOWH032 did not recapitulate the neuroprotection observed with the knockout of the genes using CRISPR-Cas9. This could be explained by multiple reasons. Firstly, we only tested a few concentrations and one time point. Longer treatments might be required to detect an effect. We were replacing the media every two days, but depending on the inhibitors’ half-life, more frequent media changes might be required. Also, the efficiency of the drugs to inhibit the proteins in our system was not tested. In this study, we used Tun as a chemical inducer of ER stress. Further experiments are required to test whether the identified hits provide neuroprotection against other chemical inducers or models of ER stress.

KAT2B was one of the identified and validated hits, whose pharmacological inhibition, using L-Moses, attenuated neuronal cell death, reduced CHOP levels, and reversed the transcriptional changes caused by Tun treatment in both cortical and dopaminergic neurons. KAT2B possesses intrinsic histone acetyltransferase activity^27^. Histone acetylation generally promotes gene expression and therefore supports many aspects of learning and memory^35^. However, KAT2B appears to function atypically in AD. While KAT2B activation is beneficial for memory in normal rodents^36,37^, knockout or inhibition of KAT2B attenuates AD-like cognitive deficits in Aβ-treated rodents^38,39^. Specifically, *KAT2B* knockout mice are resistant to Aβ_25-35_-mediated learning and memory deficits, as well as neurotoxicity of CA1 pyramidal cells in the hippocampus^38^. Similarly, pharmacological inhibition of KAT2B, using C-30-27 compound, improved the cognitive deficits and the damaged cholinergic system in rats treated with Aβ, by reducing the neuroinflammatory response^39^. In addition, Creighton et al. suggested an age-related bidirectional role for KAT2B in AD, where activation is initially beneficial for memory, but becomes detrimental as the disease progressed^40^. Interestingly, KAT2B was shown to directly control UPR gene expression by occupying their promoters in murine pancreatic cells^41^. Li and colleagues showed that KAT2A, a KAT2B paralogue, physically interacts with CHOP to modulate ER-stress induced TNFRSF10A and TNFRSF10B expression and apoptosis^42^. These data agree with our findings that inhibition of KAT2B under ER stress is beneficial for both cortical and dopaminergic neurons, by attenuating transcriptional changes caused by Tun treatment.

In addition to acetylating histones and therefore controlling transcription, KAT2B was shown to acetylate other proteins, including transcription factors and cytoskeletal components^43^. KAT2B contains a single bromodomain, an N-terminal domain and a histone acetyltransferase domain. L-Moses, the inhibitor used in this study, is as a potent, selective (> 4500-fold selective over BRD4), and cell-active KAT2B bromodomain inhibitor^10^. It is therefore not surprising that, in our study, L-Moses attenuated transcriptional changes caused by Tun, but not the lysine acetylation changes on proteins. Importantly, L-Moses showed no cytotoxicity in peripheral blood mononuclear cells, displayed good kinetic solubility and permeability in cells and was metabolically stable in the human and mouse liver^10^, important properties for a potential therapeutic drug.

We demonstrated that KAT2B inhibition by L-Moses is neuroprotective against Tun in both cortical and dopaminergic neurons. However, electrophysiology is required to investigate how Tun affects the function of these neurons and whether L-Moses can reverse any Tun-mediated effects in neuronal function, in addition to prevent their cell death.

In summary, we provided evidence that KAT2B knockout provides resistance against Tun-mediated neuronal cell death in our iPSC-derived cortical neurons. In addition, pharmacological inhibition of KAT2B, using L-Moses, reduced ER stress and attenuated neuronal cell death, at least in part by reducing the transcriptional changes caused by Tun in both cortical and dopaminergic neurons. Together our data highlight a great therapeutic potential for KAT2B and its inhibitor L-Moses against ER stress mediated defects in AD and PD.

## Supplementary Information

Supplementary Information 1.

Supplementary Information 2.

Supplementary Information 3.

Supplementary Information 4.

Supplementary Information 5.

Supplementary Information 6.

Supplementary Information 7.

Supplementary Information 8.

## Data Availability

The mass spectrometry proteomics data have been deposited to the ProteomeXchange Consortium (http://proteomecentral.proteomexchange.org/cgi/GetDataset) via the PRIDE^44^ partner repository with the dataset identifier PXD038395.
