# Supplementary material for: CRISPR-Cas9 genetic screen leads to the discovery of L-Moses, a KAT2B inhibitor that attenuates Tunicamycin-mediated neuronal cell death
Source: Sci Rep. 2023 Mar 9;13:3934. doi: 10.1038/s41598-023-31141-6 (PMC9998435; doi:10.1038/s41598-023-31141-6)
Supplement: Supplementary file 1 — Supplementary Information 1. [file 41598_2023_31141_MOESM1_ESM.docx]

**Supplementary Methods**

**Druggable genome pool library design and cloning**

Exonic CRISPR sites for each of the 4401 druggable genes were selected as previously described (1). The resulted library, in which most genes had six sgRNAs, consisted of 26306 sgRNAs. 1000 non-targeting sgRNAs were also included as controls. Full list of sgRNAs of the druggable genome library is shown in **Supplementary Table 1**. Single strand oligo pools were synthesized by Custom Array and cloned into the *BbsI* site of pLV-PB-U6gRNA(*BbsI*)-PGKpuro2ABFP (Lenti-PB) plasmid using GIBSON cloning (NEB) as previously described (1,2).

**Lentivirus production and transduction**

Lentivirus production and transduction were performed as previously described (3), with some modifications. For the pooled druggable library, HEK293FT cells were plated on Poly-D-Lysine coated T175 flasks. When reached 60-80% confluency, cells were transfected with 13.75 μg lentiviral plasmids, 10.5 μg pPAX2 and 6.87 μg pMD2.G using Lipofectamine LTX with PLUS reagent (ThermoFisher Scientific). The medium (DMEM/F12) was replaced 24h post transfection and viral supernatant was harvested 72h post transfection. Viral preparations were concentrated by overnight centrifugation at 6000 g at 4°C, aliquoted and stored at -80°C. Lentiviral titer was determined by transducing d4 neurons with a series dilution of the viral preparations.

For the arrayed screen, individual lentiviral plasmids containing a sgRNA were transfected into HEK293FT cells in a 96-well plate format. DMEM/F12 was replaced by 300 μl of Neurobasal supplemented with B27, Glutamax and Pen/Strep, 24 h post transfection. The viral preparations were collected 72 h post transfection, centrifuged at 300 g for 5 minutes to pellet dead cells, aliquoted into fresh 96-well plates and stored at -80°C. The viral preparations of the three sgRNAs for each gene were pooled prior to the transduction and added to the media at 1:10 ratio. Three transductions for each gene were performed as replicates and the experiment (lentiviral production and transduction) was performed twice.

**CRISPR-Cas9 pooled screen**

D4 neurons were transduced with the sgRNA library at multiplicity of infection (MOI) of 0.3, as measured by BFP expression from the lentiviral vector 3 days post infection, with approximately 350x coverage per library element. Three lentiviral transductions were performed and used as replicas. D14 neurons were treated with 100 nM Tun for 7 days. On d21, single cell suspensions were centrifuged at 90 g for 10 min to remove dead cells. Cells were centrifuged again at 250 g for 5 min to pellet live cells. DNA extraction was performed by incubating cell pellets in lysis buffer (50 mM Tris pH8, 100 mM NaCl, 10 mM EDTA pH8, 1% SDS, 0.5 mg/ml proteinase K) for 4 hours at 55°C. sgRNA-encoding regions were amplified and sequenced on Illumina NextSeq 550 as previously described (4).

After sequencing, 21nt sequencing reads were exported using bcl2fastq v2.2.0 and counted by converting them to k-mers and mapping them to our CRISPR library 20nt gRNA sequences. Reads without exact match in the CRISPR library were discarded. Samples were inspected for proper sgRNA infection and coverage. With all the samples passing QC, MAGeCK RRA (5) was used to perform gene essentiality and enrichment inference. Hits were called based on the “pos|p-value” in gene summary (pos|p-value < 0.05), defined as the raw p-value (using permutation) of this gene in positive selection. We used p-value as our cut off to be more inclusive in the analysis and selection of hits for arrayed validation.

For the arrayed-screen validation, 38 genes that had at least three sgRNAs significantly enriched in the Tun versus the untreated samples were selected. The three sgRNAs with the higher p-value based on the MAGeCK analysis were re-cloned in a 96-well arrayed format as previously described (1). The sgRNA IDs and oligonucleotide sequences are shown in **Supplementary Table 1**.

**BRB-seq**

RNA was extracted using Macherey-Nagel^TM^ Mini Spin Column kit (ThermoFisher Scientific, #12373368), quantified using Qubit, and quality-checked using Agilent TapeStation (RIN: 8-9.5). 100 ng of total RNA (or the maximum available) was used as input. Libraries had a median size of 520-750, based on Agilent TapeStation. Library concentration was measured using the NEBNext Library Quant Kit for Illumina (New England Biolabs, cat # E7360L). Libraries were run on the Illumina NextSeq 550, using the NextSeq 500/550 High Output Kit v2.5 (75 Cycles) (Illumina, #20024906) at a concentration of 1.6 pM. The run setup was as follows: Paired end, Read 1 = 28 cycles, Index 1 = 8 cycles, Read 2 = 56 cycles.

After sequencing, a single unified FastQ file with all the sequencing reads was exported using bcl2fastq v2.2.0. The reads were demultiplexed according to the first 12 nt of Read 1 into individual sample FastQ files which contained the Read 2 56 nt sequence and the Read 1 sample sequence and a 13nt unique molecular identifier (UMI) appended to Read name. These samples were individually mapped to the GRCh38 human genome using the STAR aligner v2.7.3 (6). After mapping, a custom python script was used to filter out the duplicate (gene, UMI) mRNAs per sample. These unique mRNA molecules were aggregated in a count vector and merged into a unified count table.

For differential expression analysis, we used DESeq2 v1.36.0 (7) and included only samples with more than 100,000 mRNAs. This process excluded three samples from the analysis. The formula used for the DESeq2 model was '~clone + ER*treatment' where ‘clone’ is the cell line clone, ‘ER’ indicates Tun-treatment and ‘Treatment’ indicated L-Moses. Gene Ontology enrichment analysis was performed using Metascape (8).

**Liquid chromatography and Mass spectrometry**

Four conditions of cortical neurons were pelleted on d18: a) Unt: untreated control neurons, b) Tun: neurons treated for 4 days with 100 nM Tun, c) Tun+L-Moses: neurons pre-treated with 12.5 μM L-Moses on d12, followed by co-treatment with 100 nM Tun for 4 days, and d) L-Moses only: treated with 12.5 μM L-Moses for 6 days. Four different clones (NGN2 OPTi-OX, NGN2 OPTi-OX expressing Cas9 and the two homozygote CHOP reporter NGN2 OPTi-OX lines) were processed as biological replicas.

The cell pellets were lysed with dissolution buffer composed of 100mM triethylammonium bicarbonate (Sigma, #T4708), 1% sodium deoxycholate (SDC), 10% isopropanol, 50 mM NaCl and protease and phosphatase inhibitors (Halt, #78441). A universal nuclease (Pierce, #88700) was added to each sample followed by 15 min incubation at room temperature and brief probe sonication. The protein concentration was measured with the Bradford assay (Bio-Rad, Quick StartTM); 300 μg total protein per sample were reduced and alkylated simultaneously by adding tris-2-carboxyethyl phosphine (ΤCEP, Sigma) at a final concentration of 5 mM, and freshly prepared iodoacetamide at a final concentration of 10 mM followed by 1 h incubation at room temperature in the dark. Samples were digested overnight at 37℃ with trypsin (Pierce #90058) and peptides were labelled with the TMTpro-16plex reagents (1mg per sample) according to manufacturer’s instructions (Thermo Scientific). After labelling, sample was acidified with 20 µl formic acid followed by centrifugation at 10,000 rpm for 5 min to remove SDC. The TMT mixture was fractionated on a Dionex UltiMate 3000 system at high pH (Buffer A: 20mM ammonium hydroxide, Buffer B: 90% ACN, 20mM ammonium hydroxide) using the X-Bridge C18 column (3.5μm 2.1x150mm, Waters). Fifteen fractions were collected and 10% was transferred on different tubes for the TMT whole-proteome analysis. An orthogonal pooling was performed for the remaining amount to end-up with six fractions for the acetylome enrichment. The acetylome enrichment was performed according to manufacturer’s instructions (PTMScan HS Acetyl-Lysine Motif kit #46784). 60 µl of beads were used for the enrichment; after beads-antibody washes, beads-antibody were resuspended in 120 µl 1x HS IAP Bind Buffer and 20 µl was added to each fraction, followed by 2h incubation at room temperature. Acetylated peptides were eluted twice with 50 µl of 0.15% trifluoroacetic acid. The acetylome and whole proteome analyses were performed on a Dionex UltiMate 3000 UHPLC system coupled with the nano-ESI Fusion-Lumos (Thermo Scientific) mass spectrometer. Mobile phase A was composed of 0.1% formic acid and mobile phase B was composed of 80% ACN, 0.1% formic acid. For the whole proteome analysis, the MS2 scans were performed in the ion trap with collision energy 32%. Peptides were isolated in the quadrupole with isolation window 0.7 Th. The 10 most intense fragments were selected for Synchronous Precursor Selection (SPS) HCD-MS3 analysis with MS2 isolation window 2.0 and HCD collision energy 50%. For the acetylome analysis, the MS2 scans were performed in the orbitrap with collision energy 36%. Peptides were isolated in the quadrupole with isolation window 1.2 Th at 50,000 resolution. Raw data were processed with the SequestHT search engine on Proteome Discoverer 2.4 software and searched against a Uniprot database containing reviewed human protein entries. The parameters for the SequestHT node were as follow: Precursor mass tolerance 20 ppm, fragment mass tolerance 0.5 Da (or 0.02 Da for acetylome analysis), dynamic modifications were oxidation of M (+15.995Da), deamidation of N, Q (+0.984Da) and static modifications were TMTpro at any N-Terminus or K (+304.207 Da) and carbamidomethyl at C (+57.021 Da). For the acetylome analysis dynamic modifications were oxidation of M (+15.995Da), deamidation of N, Q (+0.984Da), TMTpro at K (+304.207Da) and acetyl at K or protein N-terminus (+42.011 Da) and static modifications were TMTpro at any N-Terminus and carbamidomethyl at C (+57.021Da). IMP-ptmRS node was used for acetyl-site localisation. The consensus workflow included TMT signal-to-noise (S/N) calculation and the level of confidence for peptide identifications was estimated using the Percolator node with decoy database search. Strict FDR was set at q-value<0.01.

Data processing, normalization, and statistical analysis of the peptide intensities were carried out using the qPLEXanalyzer (9) package from Bioconductor. In the whole-proteome analysis, peptide intensities were normalized using median scaling, and protein level quantification was obtained by the summation of the normalized peptide intensities. The acetylome analysis was performed at the modification (Acetyl) site level. Firstly, acetylated peptide intensities were normalized using median scaling. Then, the normalized dataset was transformed to represent unique modified sites of each protein by merging (summing intensity of) duplicated sites coming from the same protein. Thereafter, a statistical analysis of differentially regulated proteins and sites respectively was carried out using the Limma method. Multiple testing correction of p-values was applied using the Benjamini-Hochberg method to control the FDR. The 500 hits with the highest LFC were selected from each group for GO analysis using Metascape.

References

1. Metzakopian E, Strong A, Iyer V, Hodgkins A, Tzelepis K, Antunes L, et al. Enhancing the genome editing toolbox: genome wide CRISPR arrayed libraries. Sci Rep. 2017 22;7(1):2244.

2. Shalem O, Sanjana NE, Hartenian E, Shi X, Scott DA, Mikkelson T, et al. Genome-Scale CRISPR-Cas9 Knockout Screening in Human Cells. Science. 2014 Jan 3;343(6166):84–7.

3. Joung J, Konermann S, Gootenberg JS, Abudayyeh OO, Platt RJ, Brigham MD, et al. Genome-scale CRISPR-Cas9 knockout and transcriptional activation screening. Nat Protoc. 2017 Apr;12(4):828–63.

4. Koike-Yusa H, Li Y, Tan EP, Velasco-Herrera MDC, Yusa K. Genome-wide recessive genetic screening in mammalian cells with a lentiviral CRISPR-guide RNA library. Nat Biotechnol. 2014 Mar;32(3):267–73.

5. Li W, Xu H, Xiao T, Cong L, Love MI, Zhang F, et al. MAGeCK enables robust identification of essential genes from genome-scale CRISPR/Cas9 knockout screens. Genome Biol. 2014 Dec;15(12):1–12.

6. Dobin A, Davis CA, Schlesinger F, Drenkow J, Zaleski C, Jha S, et al. STAR: ultrafast universal RNA-seq aligner. Bioinformatics. 2013 Jan 1;29(1):15–21.

7. Love MI, Huber W, Anders S. Moderated estimation of fold change and dispersion for RNA-seq data with DESeq2. Genome Biol. 2014;15(12):550.

8. Zhou Y, Zhou B, Pache L, Chang M, Khodabakhshi AH, Tanaseichuk O, et al. Metascape provides a biologist-oriented resource for the analysis of systems-level datasets. Nat Commun. 2019 Apr 3;10(1):1523.

9. Papachristou EK, Kishore K, Holding AN, Harvey K, Roumeliotis TI, Chilamakuri CSR, et al. A quantitative mass spectrometry-based approach to monitor the dynamics of endogenous chromatin-associated protein complexes. Nat Commun. 2018 Jun 13;9(1):2311.

**Supplementary Figures**

**
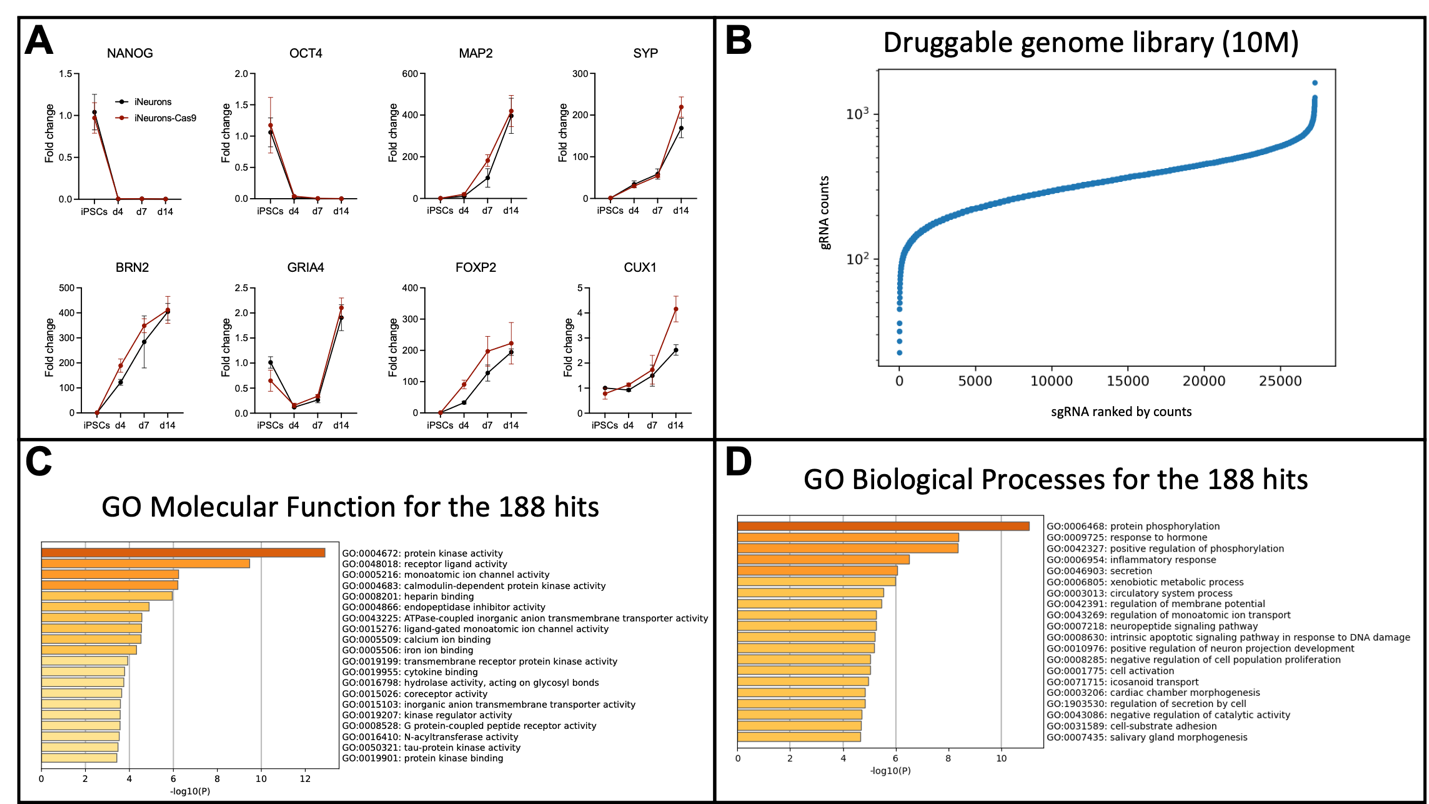
**

**Supplementary Fig. 1: Druggable genome CRISPR screen.** (A) qPCR analysis of key pluripotent (*NANOG*, *OCT2*), pan-neuronal (*MAP2*, *SYP*), forebrain (*BRN2*) and cortical (*GRIA4*, *FOXP2*, *CUX1*) markers in iNeurons (black) and iNeurons-Cas9 (red), showing that Cas9 does not affect cortical differentiation. (B) Representation of the sgRNA plasmid library. 27000 sgRNAs are shown and their counts detected in ascending order. Total library size is projected to 10 million reads. (C) Gene ontology (GO) enrichment analysis for Molecular Function and (D) Biological processes for the 188 hits identified from the pooled screen.


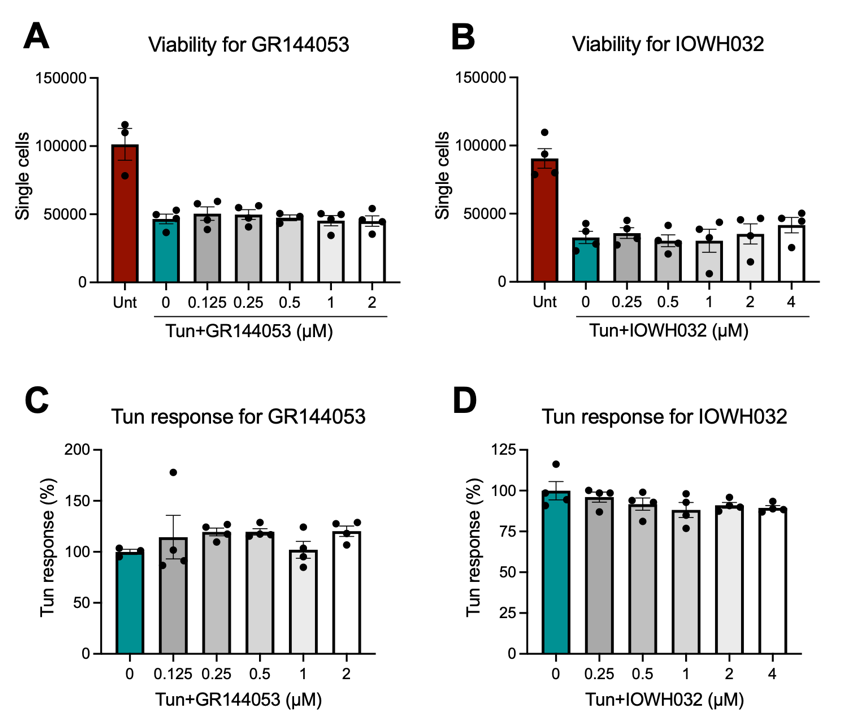


**Supplementary Fig. 2:** **GR144053 and IOWH032 does not attenuate Tun-mediated effects.** (A-B) Pharmacological inhibition of ITGA2B and CFTR, by GR144053 and IOWH032, does not attenuate Tun-mediated cell death. (C-D) The Tun-mediated increase in CHOP levels is not affected by treatment with GR144053 or IOWH032.

**
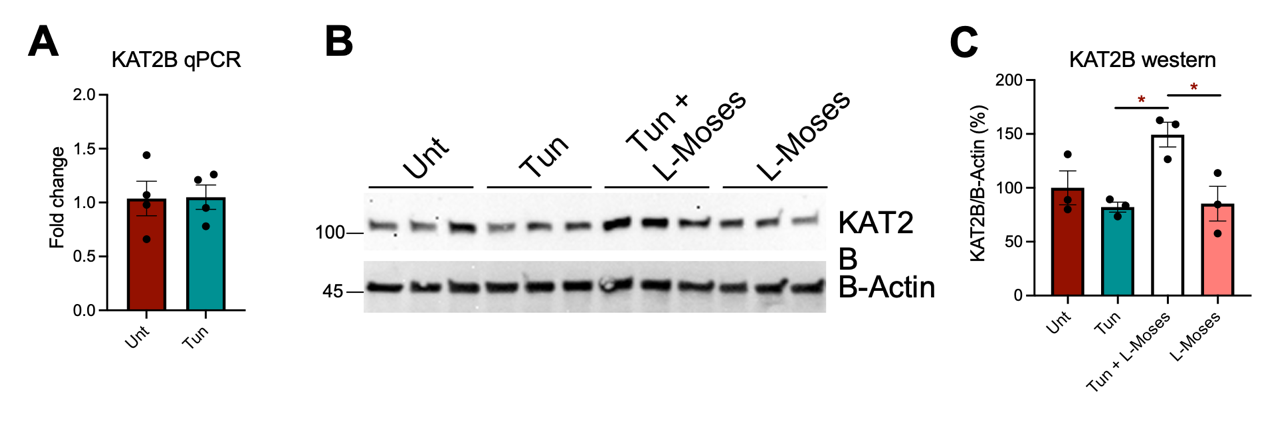
**

**Supplementary Fig. 3: KAT2B levels are not affected by Tun.** (A) *KAT2B* mRNA expression is not affected by Tun treatment. (B-C) Western blot showing increase in KAT2B expression only following Tun+L-Moses treatment. Original blots are presented in Supplementary Fig. 7.


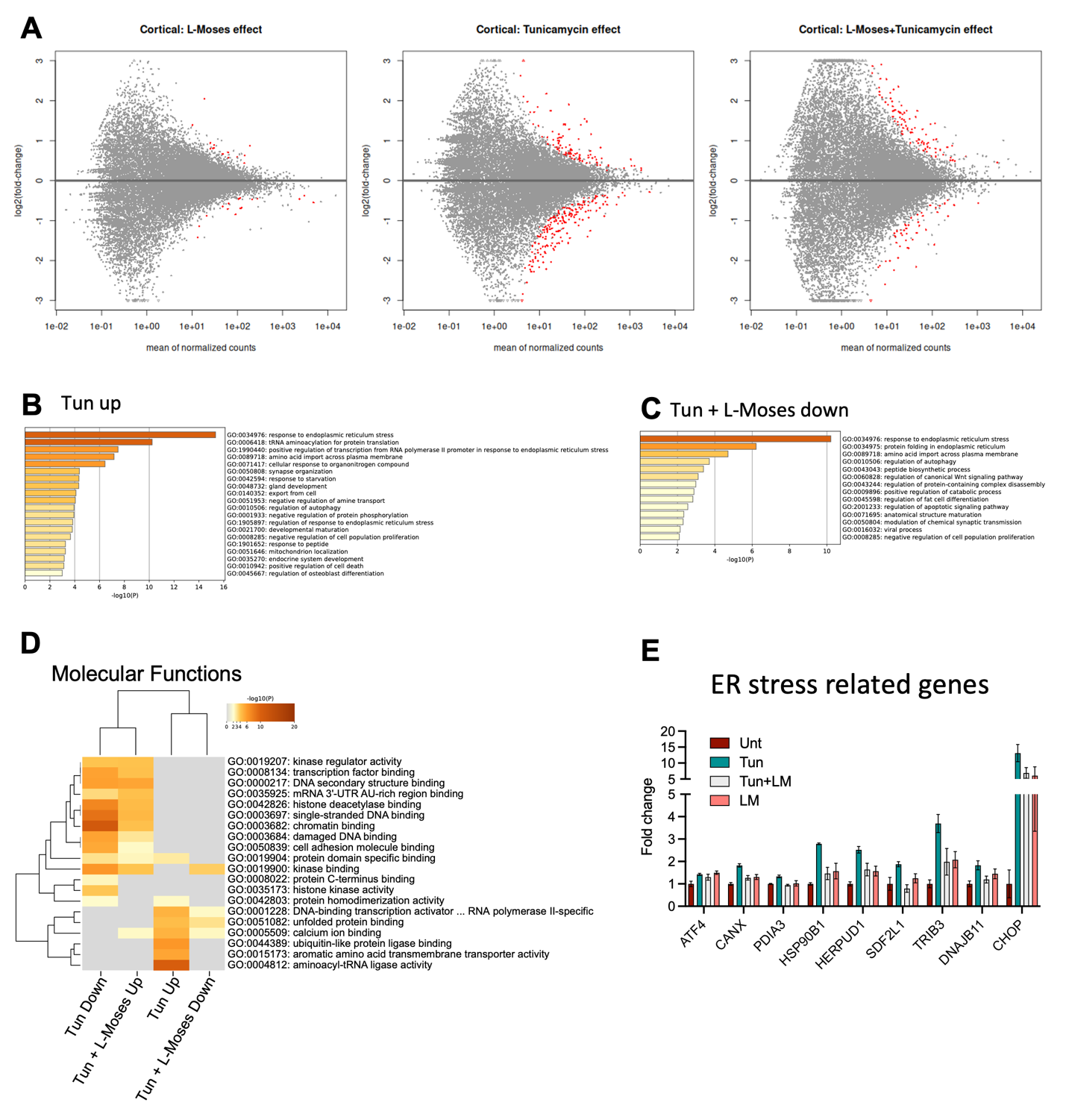


**Supplementary Fig. 4: BRB-seq data of cortical neurons.** (A) MA plots showing significantly differentially expressed genes (red dots) as a result of L-Moses, Tun or combined Tun and L-Moses treatments. (B) GO Biological processes enriched in genes upregulated by Tun treatment compared to untreated samples. (C) GO Biological processes enriched in genes downregulated by Tun+L-Moses when compared to Tun alone. (D) Hierarchical clustering of GO Molecular Functions terms upregulated or downregulated in Tun versus Tun+L-Moses in cortical neurons. (E) Plot showing the fold change in gene expression under the four different experimental conditions for genes involved in ER stress, as observed by BRB-seq.


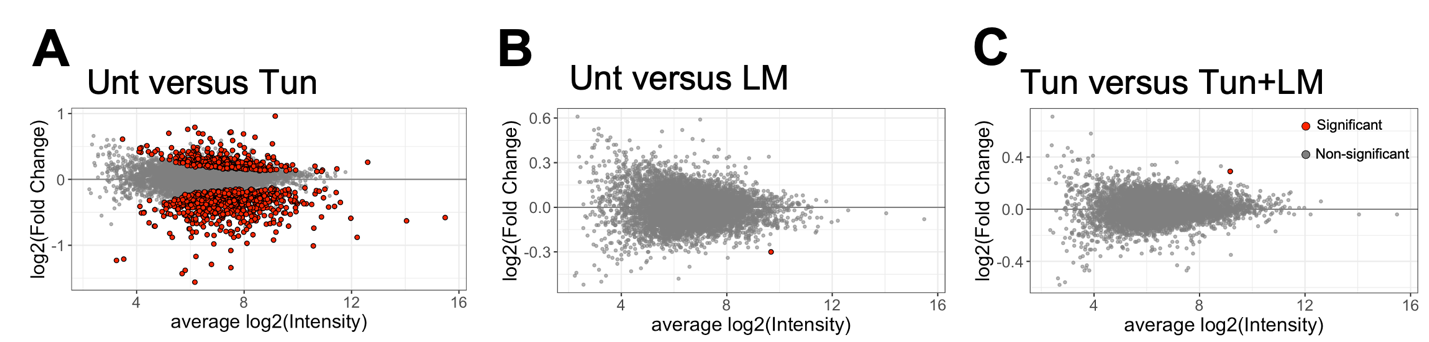


**Supplementary Fig. 5: Acetylome analysis of cortical neurons.** (A) MA plots showing significantly differentially expressed lysine acetylated sites between untreated and Tun-treated cortical neurons in red. (B) MA plot showing significantly differentially expressed lysine acetylated sites between Unt and L-Moses treated cortical neurons in red. (C) MA plot showing significantly differentially expressed lysine acetylated sites between Tun and Tun+L-Moses-treated cortical neurons in red.

**
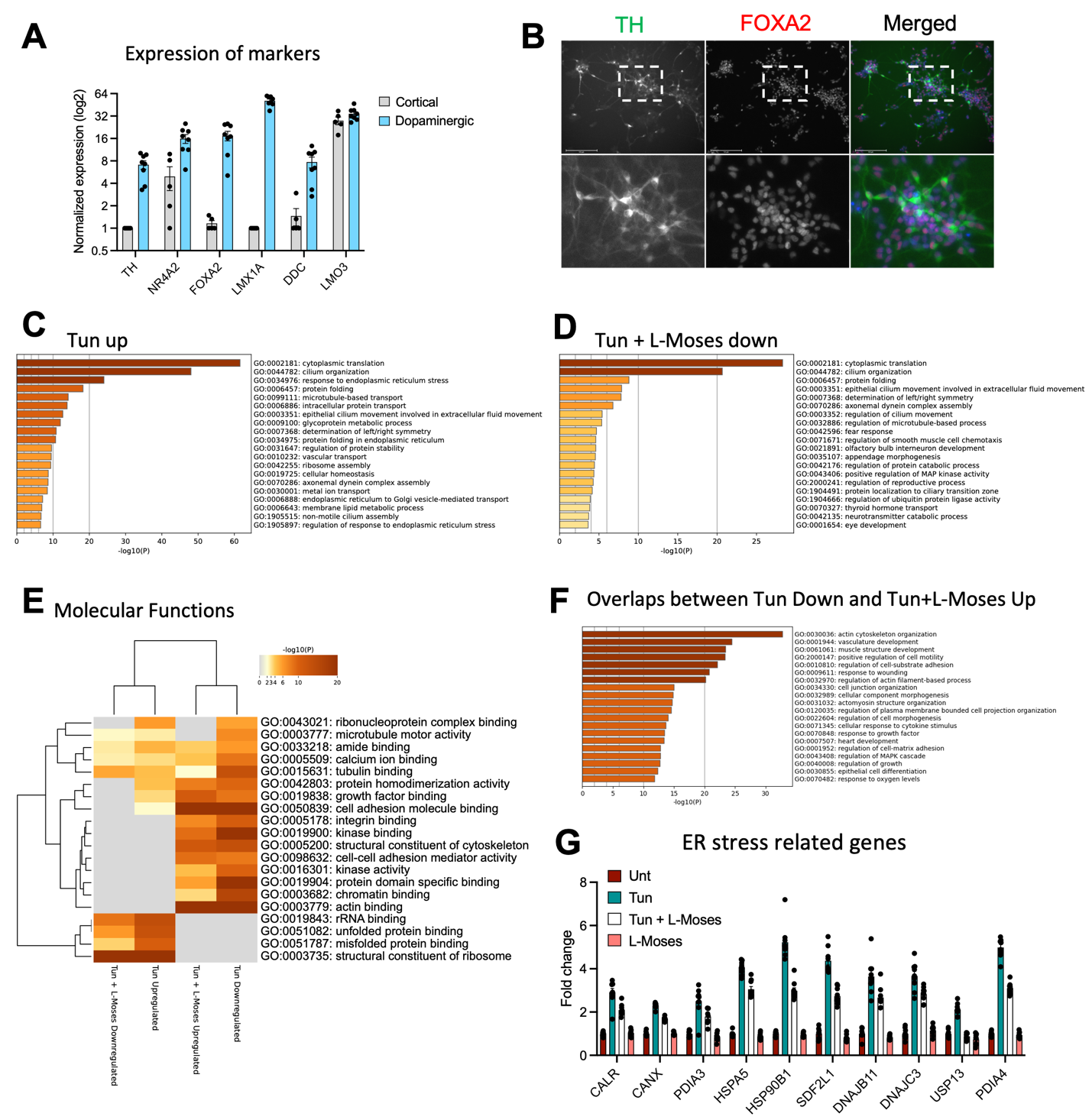
**

**Supplementary Fig. 6: BRB-seq in dopaminergic neurons.** (A) Expression levels of key dopaminergic markers in cortical (grey) and dopaminergic (blue) neurons, by BRB-seq. (B) Immunofluorescence showing expression of TH and FOXA2 in dopaminergic neurons. (C) GO Biological processes enriched in genes upregulated by Tun treatment compared to untreated samples. (D) GO Biological processes enriched in genes downregulated by Tun+L-Moses when compared to Tun alone. (E) Hierarchical clustering of GO Molecular Functions terms upregulated or downregulated in Tun versus Tun+L-Moses in dopaminergic neurons. (F) GO Biological Processes terms enriched in the genes that overlap between the ones downregulated by Tun and upregulated by Tun+L-Moses. (G) Plot showing the fold change in gene expression under the four different experimental conditions for genes involved in ER stress, as observed by BRB-seq.

**
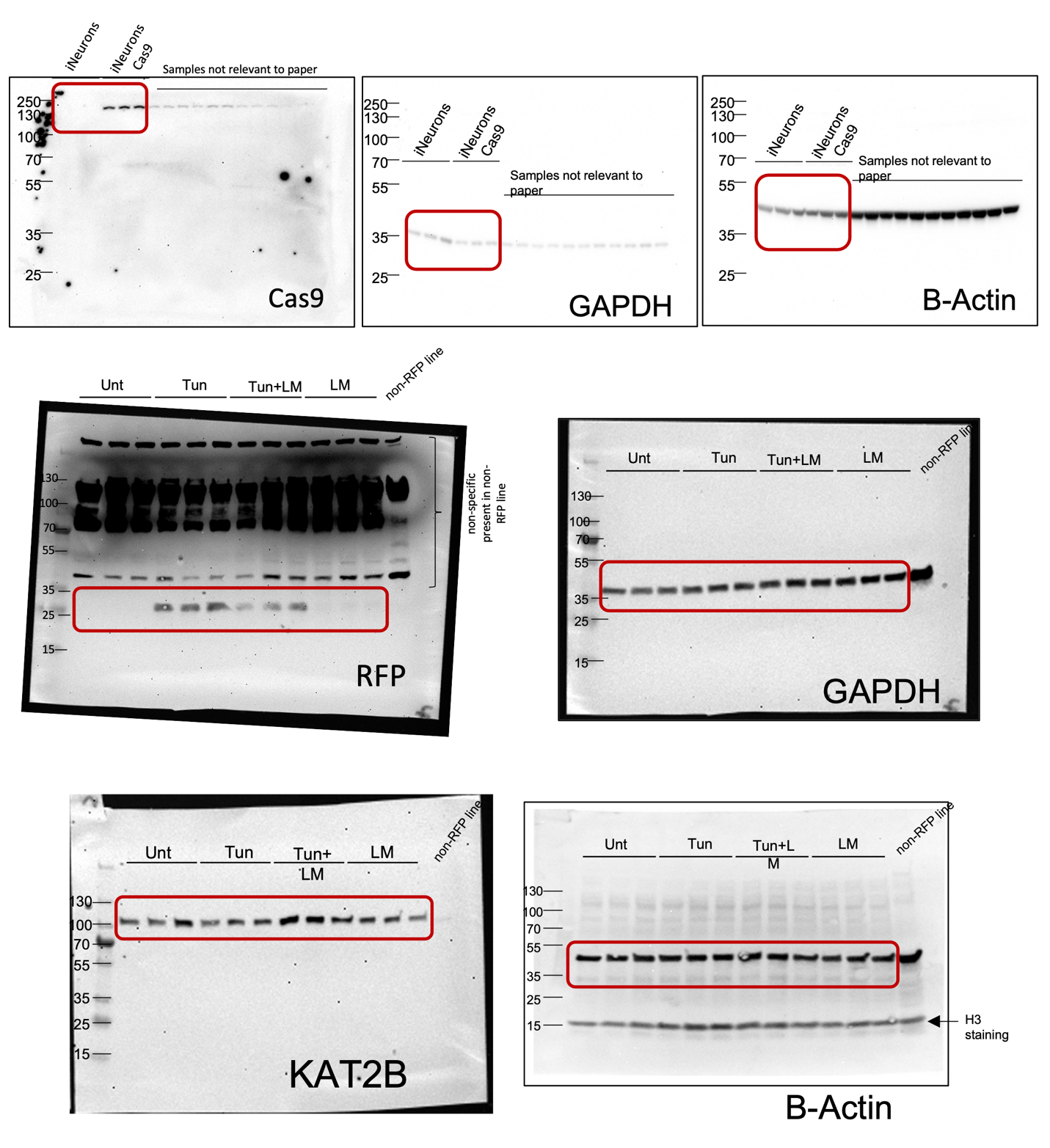
**

**Supplementary Fig. 7: Original western blots.** Cropped areas displayed in main figures are marked within red lines.
